# Low voltage control of exchange coupling in a ferromagnet-semiconductor quantum well hybrid structure

**DOI:** 10.1038/s41467-019-10774-0

**Published:** 2019-07-01

**Authors:** V. L. Korenev, I. V. Kalitukha, I. A. Akimov, V. F. Sapega, E. A. Zhukov, E. Kirstein, O. S. Ken, D. Kudlacik, G. Karczewski, M. Wiater, T. Wojtowicz, N. D. Ilyinskaya, N. M. Lebedeva, T. A. Komissarova, Yu. G. Kusrayev, D. R. Yakovlev, M. Bayer

**Affiliations:** 1 Ioffe Institute, Russian Academy of Sciences, 194021 St. Petersburg, Russia; 2 Experimentelle Physik 2, Technische Universität Dortmund, D-44227 Dortmund, Germany; 3 Institute of Physics, Polish Academy of Sciences, PL-02668 Warsaw, Poland; 4 International Research Centre MagTop, Institute of Physics, Polish Academy of Sciences, PL-02668 Warsaw, Poland

**Keywords:** Spintronics, Ferromagnetism

## Abstract

Voltage control of ferromagnetism on the nanometer scale is highly appealing for the development of novel electronic devices with low power consumption, high operation speed, reliable reversibility and compatibility with semiconductor technology. Hybrid structures based on the assembly of ferromagnetic and semiconducting building blocks are expected to show magnetic order as a ferromagnet and to be electrically tunable as a semiconductor. Here, we demonstrate the electrical control of the exchange coupling in a hybrid consisting of a ferromagnetic Co layer and a semiconductor CdTe quantum well, separated by a thin non-magnetic (Cd,Mg)Te barrier. The electric field controls the phononic ac Stark effect—the indirect exchange mechanism that is mediated by elliptically polarized phonons emitted from the ferromagnet. The effective magnetic field of the exchange interaction reaches up to 2.5 Tesla and can be turned on and off by application of 1V bias across the heterostructure.

## Abstract (web summary)

Manipulating magnetic orders by electric fields promises energy efficient spintronic applications. Here the authors demonstrate electrical control of s-p exchange interaction in a ferromagnet-semiconductor quantum well structure due to the phononic ac Stark effect.

## Introduction

Nowadays the demand for control of ferromagnetism on the nanometer scale is met by the methods of spin-transfer torque or spin–orbit torque, both based on locally controlled magnetization reversal by a high-density current of ~10^6^ A cm^−2 ^^1^. However, more promising in terms of energy costs is the use of an electric field, instead of electrical current or magnetic field, which would allow fast voltage control of magnetism^2^. This type of control was realized, for instance, for the low-temperature magnetic semiconductors (In,Mn)As and (Ga,Mn)As^3^, and more recently significant progress in that direction was achieved in various materials. Examples are the coercive force in multiferroics^4^, the magnetic anisotropy in ultrathin Fe/MgO^2^ and the magnetic order in ferromagnetic–ferroelectric structures^5^. The most intriguing idea for tuning magnetic properties is based on the control of the exchange interaction causing the magnetism (the strongest spin–spin interaction) through varying the carrier wavefunction overlap in a thin magnetic layer. However, this requires application of rather strong electric fields of ~10^7^ V cm^−1 ^^4^. Therefore, alternative concepts for magnetism control that allow one to use low electric fields at elevated temperatures, are actively pursued. Moreover, an additional requirement for applications is the integration of the magnetic system into an electronic device compatible with current semiconductor technology.

Hybrid systems that combine thin ferromagnetic (FM) films with semiconducting (SC) layers are promising for unifying magnetism and electronics, which may allow all-in-one-chip solutions for computing. To that end, the hybrids need to show magnetic order as a ferromagnet, while remaining electrically reconfigurable as a semiconductor^6,7^. By now, ferromagnetic proximity effects were revealed optically^8,9^ and electrically^10^. Further, electrical measurements using the anomalous Hall effect demonstrated that the p–d exchange interaction of the magnetic atoms in a ferromagnetic film (the d-system) with a two-dimensional hole gas (2DHG, the p-system) in a semiconductor quantum well (QW) induces an equilibrium spin polarization of the QW holes^10^. Optical studies^8,9^ showed polarized photoluminescence (PL) from the QW located a few nanometers apart from the FM. However, care has to be exercised in the interpretation of the FM proximity effect, when electrons and holes are present in non-equilibrium: a previous study^11^ had demonstrated that under optical excitation an alternative mechanism exists involving spin-dependent capture of charge carriers from the SC into the FM, representing a dynamical spin polarization effect in contrast to the exchange-induced equilibrium polarization. The wavefunction engineering strategy based on electric field control of the overlap of charge carrier wavefunctions in a quantum well with localized d-electrons had been proposed in ref. ^12^. Control of the ferromagnetism in the low-temperature FM (In,Fe)As was experimentally demonstrated in ref. ^13^. All these mechanisms are based on wavefunction overlap and, therefore, lead to short-range proximity effects.

A conceptually different type of long-range FM proximity effect was reported recently for a hybrid Co/CdTe structure^14^. It is manifested by the spin polarization of holes bound to shallow acceptors in a nonmagnetic CdTe quantum well due to an effective long-range p–d exchange interaction that is not related to the penetration of the electron wavefunction into the FM layer. This interaction was conjectured to be mediated by elliptically polarized phonons with energy close to the magnon–phonon resonance in the FM. The long-range exchange constant was directly measured by spin-flip Raman scattering (SFRS) in ref. ^15^. However, no electric control of this exchange coupling has been demonstrated so far.

Here, we show that application of an electric field across the structure changes the strength of the long-range p–d exchange coupling between the FM and the SC, namely between the holes bound to acceptors in the quantum well. The coupling is controlled by the band bending in the quantum well region, becoming most efficient in the case of flat bands. The effective magnetic field of the exchange interaction reaches 2.5 T and can be turned on and off by application of ~1 V bias across the heterostructure. The control is not related to a spatial redistribution of wavefunctions and, therefore, cannot be explained using the standard model of exchange interaction. In contrast, it can be well described in the framework of the exchange mechanism mediated by elliptically polarized phonons. The applied voltage varies the heavy–light-hole transition to which the phonons couple, bringing it in and out of resonance with the magnon–phonon resonance of the FM. Doing so, the effective exchange coupling strength in the hybrid system is tuned electrically without any power consumption, using field strengths of about 10^4^ V cm^−1^ only, which is a few orders of magnitude reduced in comparison to non-semiconductor systems. Therefore, our results pave the way for integration of electrically tunable magnetism into semiconductor electronics. Moreover, the presented electric control of the exchange coupling by elliptically polarized phonons can be implemented not only in semiconducting, but also in metallic and dielectric systems. For example, one layer of a ferromagnetic metal (FM) could emit elliptically polarized phonons, which are transmitted through a paramagnetic metal (PM) and then penetrate another FM whose magnetic state is switched thereby in such a FM/PM/FM trilayer hybrid. Further, the concept of elliptically polarized phonons is relevant beyond magnetic spintronics, because these phonons could be created without magnets, using materials with large birefringence of sound to produce the phononic analog of an optical quarter wave plate. Our work establishes an unexplored direction of helical phononics^14,16,17^.

## Results

### Ferromagnetic proximity effect in steady state

**Fig. 1:**
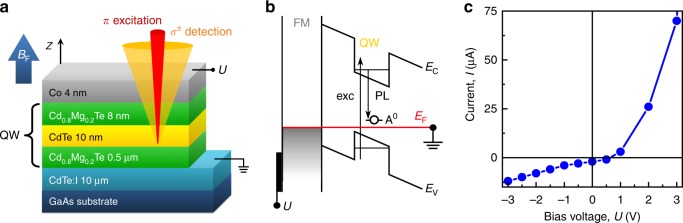
Sample characterization. **a** Schematic device architecture and geometry of the cw experiment. **b** Schematic presentation of the band diagram at *U* = 0, where *E*_V_ and *E*_C_ denote the valence and conduction bands, respectively, and *E*_F_ indicates the Fermi level. **c** Current–voltage characteristics *I*(*U*) under excitation with laser light in cw mode

The investigated Co/(Cd,Mg)Te/CdTe/(Cd,Mg)Te/CdTe:I/GaAs hybrid structure was grown by molecular-beam epitaxy on a GaAs substrate followed by a conducting CdTe:I layer (10-μm thickness, iodine-doped with donor concentration of ~10^18^ cm^−3^) as sketched in the layer-by-layer design in Fig. 1a. The QW is formed by a 10 nm CdTe layer sandwiched between layers of 0.5 μm (Cd,Mg)Te and 8 nm (Cd,Mg)Te (the spacer). On top of this structure, the 4 nm thick cobalt film was deposited. A mesa of 5 mm diameter was lithographically patterned by deep etching, so that an applied voltage drops between the Co and CdTe:I layers. Figure 1b schematically shows the band diagram of the structure. The current–voltage characteristics *I*(*U*) in Fig. 1c reflects the typical behavior of a Schottky diode shifted downwards along the *I*-axis due to the photovoltaic effect.

We study the ferromagnetic proximity effect using polarized PL spectroscopy in the continuous wave (cw) mode. The sample is excited by linearly polarized (π) light and the degree of circular polarization $\rho _{\mathrm{c}}^{\mathrm{\pi }}$ of the photoluminescence from the quantum well is detected. The value of $\rho _{\mathrm{c}}^{\mathrm{\pi }}$ does not depend on the orientation of the linear laser polarization. To magnetize the interfacial ferromagnet, which is responsible for the FM proximity effect^14^, we apply a magnetic field *B*_F_ in the Faraday geometry parallel to the growth axis of the heterostructure (*z*-axis, Fig. 1a).

**Fig. 2:**
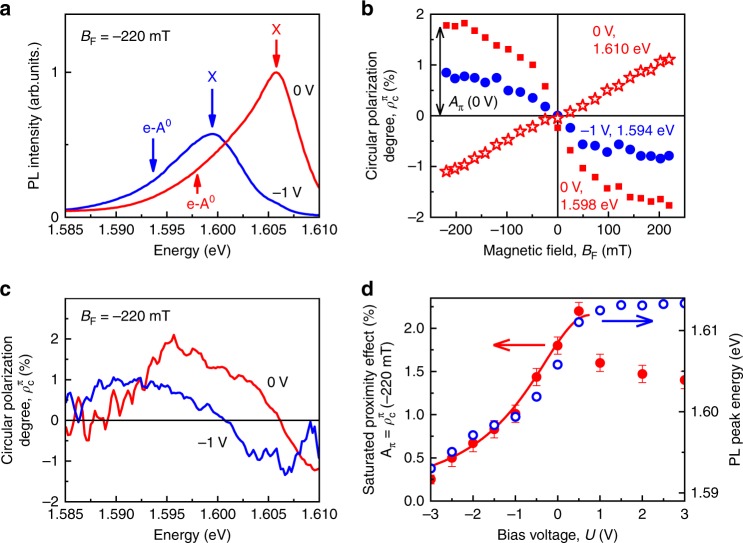
Polarization spectroscopy in the cw regime. **a** PL spectra at *U* = 0 (red) and −1 V (blue). **b** Magnetic field dependences of the circular polarization degree $\rho _{\mathrm{c}}^{\mathrm{\pi }}\left( {B_{\mathrm{F}}} \right)$ measured at *U* = 0 V, *ħω*_PL_ = 1.598 eV (red squares), *U* = −1 V, *ħω*_PL_ = 1.594 eV (blue circles), and *U* = 0, *ħω*_PL_ = 1.610 eV (red stars), using linearly polarized excitation. The arrow indicates the amplitude *A*_π_(0 V) of the FM proximity effect for *U* = 0 at 1.598 eV detection energy. **c** Spectral dependences of $\rho _{\mathrm{c}}^{\mathrm{\pi }}$ for *U* = 0 V and −1 V. **d** Amplitude *A*_π_(*U*) of the FM proximity effect (red circles) and the energy of the PL peak *ħω*_max_(*U*) (blue circles) as functions of the gate voltage. The red curve is a fit using Eq. (8) with *A* = 2.2%, *U*_0_ = 0.8 V, and *U*_1_ = 1.8 V. Excitation was done with photon energy 1.691 eV (below the band gap of the (Cd,Mg)Te barriers) using a power density of 4 W cm^−2^. In all cases the magnetic field was *B*_F_ = −220 mT (except of (**b**)) and temperature *T* = 2 K. Error bars represent standard deviations

The red curves in Fig. 2a, c correspond to zero bias, the blue ones to *U* = −1 V, all taken at *B*_F_ = −220 mT. In the PL spectrum (Fig. 2a), two features are observed. The PL band at higher photon energies (X) corresponds to the recombination of the exciton in the QW, while the low-energy tail (e-A^0^) is attributed to the recombination of an electron with a hole bound to a shallow acceptor in the QW^14^. At zero bias *U* = 0 (red curves) the photoluminescence reveals a polarization of about 2% at the acceptor band (Fig. 2c), undergoing a sign change toward the exciton emission. Application of a reverse bias (blue curves at *U* = −1 V) shifts the entire spectrum to lower energies by 7 meV due to the strong bending of the energy bands by the (static) Stark effect. Simultaneously the PL intensity decreases due to the separation of electron and hole in the QW by the electric field, leading to a reduced transition matrix element. Here the polarization degree around the acceptor emission is reduced to about 1% (Fig. 2c). Note that usage of a photo-elastic modulator operating at 50 kHz instead of a mechanically rotating quarter wave plate reduces the error bar down to 0.1%.

Next, the magnetic field dependencies of $\rho _{\mathrm{c}}^{\mathrm{\pi }}(B_{\mathrm{F}})$ were measured in the Faraday geometry for different fixed biases *U*. The FM proximity effect is assessed by the degree of circular polarization of the e-A^0^ PL versus magnetic field *B*_F_. The degree $\rho _{\mathrm{c}}^{\mathrm{\pi }}(B_{\mathrm{F}})$ saturates in the field range of 150–200 mT (red squares for *U* = 0 and blue circles for *U* = −1 V in Fig. 2b). Since the spectral positions of the emission bands are sensitive to the applied voltage *U*, the polarization was detected at the photon energy *ħω*_PL_ corresponding to the maximum polarization of the e-A^0^ transition (Fig. 2c). The magnitude of the FM proximity effect is given by the saturation polarization at the acceptor band $A_{\mathrm{\pi }} \equiv \rho _{\mathrm{c}}^{\mathrm{\pi }}$(*B*_F_ = −220 mT) which is larger for 0 V than for the reverse bias of −1 V. In contrast to the e-A^0^ transition, the polarization $\rho _{\mathrm{c}}^{\mathrm{\pi }}(B_{\mathrm{F}})$ near the exciton PL maximum depends linearly on magnetic field across the whole scanned field range without any saturation (red stars in Fig. 2b, *U* = 0, *ħω*_PL_ = 1.610 eV), independent of the applied bias *U*. The linear dependence of $\rho _{\mathrm{c}}^{\mathrm{\pi }}(B_{\mathrm{F}})$ originates from the X splitting in two lines with opposite circular polarization due to the Zeeman effect^14^. The linear *B*_F_-dependence of $\rho _{\mathrm{c}}^{\mathrm{\pi }}(B_{\mathrm{F}})$ indicates that the proximity effect is absent for the valence band holes that contribute to the exciton within its lifetime ^14^.

Figure 2d shows the dependence of the saturation polarization *A*_π_(*U*) (red circles) and the energy of the PL peak *ħω*_max_(*U*) (blue circles) as function of applied bias. The energy *ħω*_max_(*U*) increases with bias due to the reduction of the inclination of bands and consequently of the static Stark effect. The external positive bias decreases the built-in electric field. It is canceled at *U* >+0.5 V (flat band conditions) as evidenced also by a steep increase of current through the device (Fig. 1c). In this case the voltage drop is redistributed all over the structure plane (Fig. 1a), so that a voltage increase does not lead to a further band inclination. For *U* ≤ + 0.5 V the bias increases the built-in electric field and we observe a striking correlation between the voltage dependences of the magnitude of the FM proximity effect *A*_π_(*U*) and the peak position *ħω*_max_(*U*) (see Fig. 2d). In turn, for *U* *>*+0.5 V, the FM proximity effect decreases about 1.5 times, reaching the level of 1.5%. This drop is attributed to the appearance of additional holes in the valence band of the QW which contribute to the PL but have negligible p–d exchange coupling (for details see Supplementary note 1 and 2).

Here, we concentrate on the origin of the voltage dependence *A*_π_(*U*) for *U* *<* + 0.5 V. The FM proximity effect was shown^14^ to originate from the effective p–d exchange interaction $\frac{1}{3}{\mathrm{\Delta }}_{{\mathrm{pd}}}J_z$ between the interfacial FM formed at the Co/(Cd,Mg)Te interface with an out-of-plane magnetization due to the perpendicular magnetic anisotropy and the acceptor-bound QW holes with momentum projections *J*_*z*_ = ± 3/2 onto the *z-*axis. The magnetization of the Co layer is located in the plane of the structure and does not contribute to the circular polarization of the PL in weak magnetic fields^14,18^. The saturation amplitude *A*_π_ of the PL polarization is caused by the spin polarization *P*_A_ of A^0^ when the FM is completely magnetized

1 $$A_{\mathrm{\pi }} = P_{\mathrm{A}} = - \frac{{\tau _{\mathrm{A}}}}{{\tau _{\mathrm{A}} + \tau _{{\mathrm{sA}}}}}\frac{{{\mathrm{\Delta }}_{{\mathrm{pd}}}}}{{2k_{\mathrm{B}}T}}.$$

Here *τ*_A_ is the lifetime and *τ*_sA_ is the spin relaxation time of the heavy holes on acceptors, *k*_B_ is the Boltzmann constant, *Т* is the lattice temperature, and Δ_pd_ is the spin splitting of the ±3/2 levels in the effective magnetic field of the p–d exchange interaction. A positive sign of Δ_pd_ implies that the –3/2 state is energetically favorable^14^. The polarization *P*_A_ can depend on the bias *U* through four different dependencies: (1) the ratio of the times *τ*_A_/*τ*_sA_ = *f*(*U*), (2) the strength Δ_pd_(*U*) of the p–d exchange, (3) the lattice heating *T*(*U*) by electrical current, and (4) the injection of spin-polarized carriers from the FM. Heating can be excluded because the electrical power in our experiment was two orders of magnitude (<40 μW) smaller than the optical power. Heating due to the injection of hot holes and the spin injection option can be ruled out because the amplitude *A*_π_(*U*) does not follow the electric current *I*(*U*) (Fig. 1c). Time-resolved PL and spin-flip Raman scattering experiments demonstrate that the Δ_pd_(*U*) dependence is the main origin of *A*_π_(*U*).

### Electrical control of the kinetics of proximity effect

**Fig. 3:**
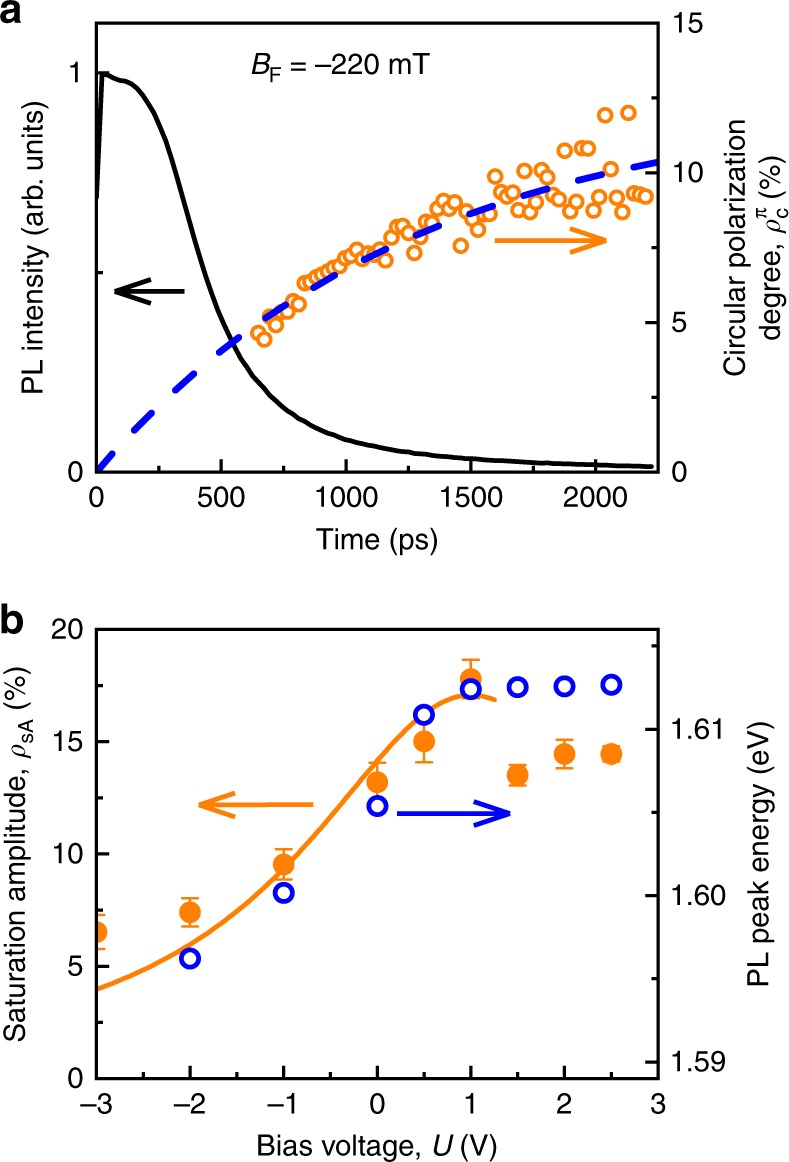
Time-resolved photoluminescence (PL). **a** The kinetics of the PL intensity (black solid line) and the degree of circular polarization of the PL (open orange circles) integrated in the spectral region of e-A^0^ at *U* = 0. Blue dashed line is a fit to the data. **b** Dependence of *ρ*_sA_(*U*) of the FM proximity effect (full orange circles) and the position of the PL peak *ħω*_max_(*U*) (open blue circles) on the bias voltage *U*. The solid curve is calculated using Eq. (8) with *A* = 17 %, *U*_0_ = 1.0 V, and *U*_1_ = 2.0 V. Error bars represent standard deviations

Time-resolved PL enables one to measure the kinetics of the PL intensity and thereby the emergence of the spin polarization induced by the magnetized FM layer (ferromagnetic proximity effect) after optical excitation of non-polarized charge carriers with linearly polarized laser pulses (Fig. 3a). In ref. ^14^, we demonstrated that the exciton PL does not reveal the FM proximity effect. The PL intensity decays much faster (a few 100 ps) than the rise of the e-A^0^ PL (~2 ns). Here, the same scenario is realized. Due to the dominant contribution of the exciton to the total PL signal especially during the first few hundred picoseconds (black curve in Fig. 3a), it is necessary to wait for 500–700 ps, until the excitons have mostly recombined, to reliably evaluate the FM proximity effect. Figure 3a (orange circles) shows the evolution of the circular polarization starting from time delays of about 700 ps. The blue dashed curve in Fig. 3a is a fit to the data according to $\rho _{\mathrm{c}}^{\mathrm{\pi }}\left( t \right) = \rho _{{\mathrm{sA}}}\left[ {1 - {\mathrm{exp}}\left( { - t/\tau_{{\mathrm{sA}}}} \right)} \right]$ with the amplitude *ρ*_sA_ = 13%, and the rise time *τ*_sA_ = 1.3 ns. This measurement was carried out at *U* = 0. From kinetic measurements of the FM proximity effect at other bias voltages one can determine the dependences *ρ*_sA_(*U*) and *τ*_sA_(*U*).

Because of the delay waiting for exciton recombination, the dependence *ρ*_sA_(*U*) can be measured more accurately than *τ*_sA_(*U*). *ρ*_sA_(*U*) has a straightforward interpretation from which Δ_pd_ can be inferred. Equilibrium occurs at delay times much longer than the spin relaxation time *τ*_sA_. Then the recombining electrons are characterized by the equilibrium spin state given by the Boltzmann distribution. Therefore, the polarization amplitude

2 $$\rho _{{\mathrm{sA}}}\left( U \right) = - \frac{{{\mathrm{\Delta }}_{{\mathrm{pd}}}(U)}}{{2k_{\mathrm{B}}T}}$$

is determined solely by the ratio of the exchange constant to the thermal energy and does not depend on the ratio of lifetime and relaxation time. Thus, the dependence *ρ*_sA_(*U*) is determined exclusively by Δ_pd_(*U*). Figure 3b shows that *ρ*_sA_(*U*) has a peak near *U* = +1 V, similar to the cw data (compare with Fig. 2d). Therefore, the time-resolved PL demonstrates that the exchange constant between the magnetic ions and the holes bound to acceptors (rather than the *τ*_A_/*τ*_sA_ ratio) is controlled by the electric field.

### Determination of the p–d exchange constant

SFRS under resonant excitation of the exciton bound to an acceptor (A^0^X) complex can be used as a reliable tool to determine directly the magnitude of the FM-induced exchange splitting^19^. In tilted magnetic field, three spin-flip processes occur as discussed in the Supplementary note 3. Using excitation with σ^–^ polarization and detection with σ^+^ cross-polarization, each of these processes results in a Stokes shift of the Raman signal by a characteristic energy. The first process is associated with a double spin flip of electron and hole with participation of an acoustic phonon. In presence of the p–d exchange interaction the Stokes shift is given by

3 $${\mathrm{\Delta }}_{\mathrm{S}}^{{\mathrm{DSF}}} = \hbar \omega _1 - \hbar \omega _2 = \mu _{\mathrm{B}}\left( {\left| {g_{\mathrm{e}}} \right| - \left| {g_{\mathrm{A}}} \right|} \right)B - {\mathrm{\Delta }}_{{\mathrm{pd}}},$$

where *ħω*_1_ and *ħω*_2_ are the energies of the incoming and scattered photons, *μ*_B_ is the Bohr magneton, *g*_e_ and *g*_A_ are the *g*-factors of the electron and the acceptor-bound hole, respectively. Another SFRS-contribution is represented by the single spin flip of the acceptor-bound hole. In the presence of the p–d exchange interaction, the corresponding Stokes shift is

4 $${\mathrm{\Delta }}_{\mathrm{S}}^{{\mathrm{SSF}}} = \hbar \omega _1 - \hbar \omega _2 = \mu _{\mathrm{B}}\left| {g_{\mathrm{A}}} \right|B - {\mathrm{\Delta }}_{{\mathrm{pd}}}.$$

The third process is associated with the spin flip of the electron in the excited A^0^X complex resulting in a Stokes shift determined solely by the Zeeman splitting, *µ*_B_|*g*_e_|*B*.

**Fig. 4:**
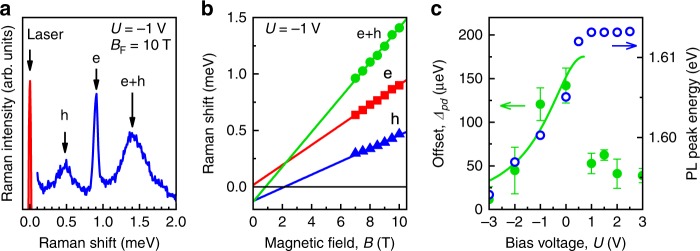
Spin-flip Raman scattering (SFRS). **a** SFRS signal under resonant excitation of the A^0^X complex at a magnetic field *B*_F_ = 10 T tilted by 20° with respect to the sample growth direction. Line h corresponds to a single acceptor hole spin-flip process, e+h is a double spin-flip process, and e is a spin flip of a conduction band electron. **b** Dependence of the Stokes shifts of the three lines on the magnetic field. **c** Dependence of the exchange constant Δ_pd_ on the applied voltage *U* for the hole bound to an acceptor (green solid circles) and the corresponding dependence of the energy of the PL peak *ħω*_max_(*U*) (blue open circles). The solid curve is calculated from Eq. (8) with *A* = 175 μeV, *U*_0_ = 0.7 V, and *U*_1_ = 1.8 V. Error bars represent standard deviations

The Raman spectrum (Fig. 4a) under resonant excitation of the A^0^X complex (1.600 eV) shows the broad SFRS line h, which is associated with the single hole spin-flip process. The e+h line corresponds to the double spin-flip process. Finally, there is also the line e, which corresponds to the electron spin flip. The sum of the energy shifts of the e and h peaks gives the energy of the e+h peak. The energies of all three SFRS lines change linearly with applied magnetic field (see Fig. 4b). However, when the magnetic field is extrapolated to zero, the Stokes shift of the line e tends to zero, while the lines h and e+h show a negative offset. This means that both SFRS lines h and e+h are influenced by the exchange interaction with the FM layer and, thus, can be used to assess the effect of gate voltage on the exchange coupling strength. The zero field offset represents a direct measurement of the exchange constant Δ_pd_. The dependence of Δ_pd_ on the applied voltage *U* is shown in Fig. 4c by the green circles. It correlates well with the voltage dependence of the PL peak position for *U* <+0.5 V. The maximum value of Δ_pd_ ≈ 150 μeV occurs in the flat band regime followed by a fast drop with increasing *U* to a value of 50 μeV, where it remains constant for *U* >+0.5 V, similar to Figs. 2d and 3b.

SFRS data at *U* *>*+0.5 V should be considered with care. As mentioned above, in this regime additional holes appear in the valence band of the QW which results in optical excitation of positively charged trions X^+^. The binding energies of the A^0^X complex and the X^+^ trion are close to each other. Therefore, the PL excitation spectra of the A^0^X and X^+^ optical transitions overlap and so do the spin flips of A^0^ and free holes. We are able to distinguish the A^0^X and X^+^ contributions as their relative intensities vary with the hole concentration, which in turn can be tuned by gate voltage. Indeed, the probabilities of A^0^X and X^+^ transitions are proportional to the concentrations of the A^0^ acceptors and valence band holes in the QW, respectively. The gate voltage controls their concentration ratio. For *U* <+0.5 V the valence band is empty of holes, so that only the A^0^X transition is excited. In this case we observe unambiguously the exchange coupling of the FM with A^0^ holes and detect the *g*-factor of A^0^ and a large offset. However, for *U* *>*+0.5 V holes appear in the QW valence band and the X^+^ transitions contribute to the spin-flip Raman process, thus changing the slope and decreasing the offset value. A detailed discussion of the *U* >+0.5 V regime is presented in the Supplementary note 2.

To conclude the SFRS results we demonstrate that the splitting value Δ_pd_ ≈ 150 μeV corresponds to an effective magnetic field of the exchange interaction of 2.5 T (the Lande *g*-factor of the hole bound to acceptor A^0^ is |*g*_A_| ≈ 1). Thus, the exchange interaction can be turned on and off by the application of ~1 V bias across the heterostructure of ≤1 μm thickness, i.e. by an electric field of about 10^4^ V cm^−1^, which is a few orders of magnitude lower than previously reported in other systems^4^.

### Electrical control of the phononic ac Stark effect

**Fig. 5:**
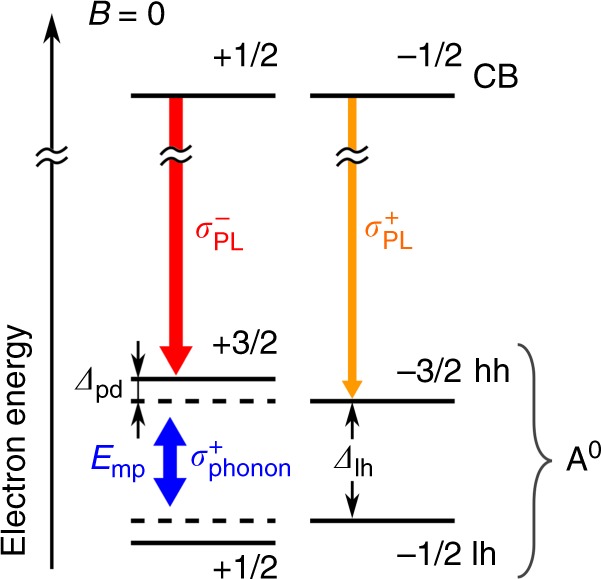
Energy scheme of the spin states. The acceptor states (A^0^) with spin projections +3/2 and +1/2 are shifted with respect to the unperturbed energy states due to the phononic ac Stark effect mediated through the elliptically polarized phonons which are emitted by the magnetized FM with preferential σ^+^ polarization. The unperturbed +3/2 and +1/2 energy states are indicated by the dashed lines. Conduction band (CB) states have spin projections ±1/2

The main finding of this work is the electric field control of the long-range exchange interaction in a hybrid ferromagnet-semiconductor structure. It can be well explained in the frame of the indirect p–d exchange mechanism mediated by elliptically polarized phonons, which represents the phononic ac Stark effect^14^. Elliptically polarized phonons exist in ferromagnets near the energy *E*_mp_ of the magnon–phonon resonance^20,21^. The FM proximity effect is based on the spin–orbit interaction of the spins of acceptor-bound holes in the QW with the nonzero angular momentum of these acoustic phonons as shown in the energy diagram of Fig. 5 in electron–electron representation.

The neutral acceptor states are split in two doublets with angular momentum projections along the *z*-axis equal to ±3/2 (heavy hole states) and ±1/2 (light-hole states). In this quadruplet in its ground state, the ±1/2 acceptor levels are populated with electrons, while the ±3/2 states are mostly empty. Interaction with circularly polarized phonons leads to an energy shift of the acceptor levels and a lifting of the doublets’ degeneracy associated with the angular momentum projection. The effect is maximal near the magnon–phonon resonance, where the energy *E*_mp_ ≈ 1 meV^20^ is close to the energy splitting Δ_lh_ ≈ 1 meV^19^ between the heavy- and light-hole acceptor states. The experimental results of ref. ^14^ demonstrate that a magnetization of the FM layer along the *z*-axis leads to negative circular polarization of the e-A^0^ optical transition, i.e. the state with +3/2 projection is shifted to higher energies with respect to the *−*3/2 level (see Fig. 5). Therefore, the unpolarized electrons from the conduction band mainly recombine into the empty +3/2 electronic states and the resulting emitted photons are σ^−^ polarized. Such a level sequence is obtained when the elliptically polarized phonons have preferential σ^+^ polarization and Δ_lh_ > *E*_mp_. Indeed, in this case the coupling is established between the electronic ground state +1/2 of the acceptor in the presence of *N* phonons and the excited state +3/2 with *N−*1 phonons. In case of Δ_lh_ > *E*_mp_ the energy levels repel each other. This is extracted from the experimental results of this work and the conclusions of ref. ^22^ where application of a static electric field *E* increases Δ_lh_, leading to an increase of the detuning Δ_lh_(*E*) − *E*_mp_ and resulting in a decrease of the interaction between the QW and the FM layer.

Similarly to the optical ac Stark effect^23^, Δ_pd_ can be calculated in second order perturbation theory

5 $${\mathrm{\Delta }}_{{\mathrm{pd}}}\left( E \right) = E_{ + 3/2} - E_{ - 3/2} = \frac{{\left| {H_{{\mathrm{ph}} - {\mathrm{h}}}} \right|^2}}{{{\mathrm{\Delta }}_{{\mathrm{lh}}}\left( E \right) - E_{{\mathrm{mp}}}}}P_{{\mathrm{phon}}}^{\mathrm{c}}.$$

Here *H*_ph–h_ is the matrix element of the interaction of the acceptor spin with the transverse acoustic phonons. Similarly to the circular polarization degree *ρ*_c_ of light, the degree of phonon circular polarization is $P_{{\mathrm{phon}}}^{\mathrm{c}} = \frac{{N_ + - N_ - }}{{N_ + + N_ - }}$, with the number *N*_+_ (*N*_−_) of right (left) circularly polarized phonons (with energy close to the magnon–phonon resonance energy). Analogously to the optical ac Stark effect, the constant Δ_pd_ is determined by the detuning Δ_lh_(*E*) − *E*_mp_ of the phonon energy *E*_mp_ (analog of the photon energy *ħω*) from the splitting Δ_lh_(*E*) (analog of the energy of the optical transition in an atom). The sign of $P_{{\mathrm{phon}}}^{\mathrm{c}}$ in the vicinity of the magnon–phonon resonance depends on the sign of the projection of the magnetization component of the interfacial ferromagnet onto the *z*-axis. The electric field *E* across the QW increases Δ_lh_(*E*) due to the static quadratic Stark effect. For small values of the electric field directed along the $\left. z \right\|[001]$ axis

6 $${\mathrm{\Delta }}_{{\mathrm{lh}}}\left( E \right) = {\mathrm{\Delta }}_{{\mathrm{lh}}}\left( 0 \right) + a_8E^2.$$

The first term Δ_lh_(0) on the right hand side in Eq. (6) corresponds to the splitting in zero electric field due to quantum confinement, while the second term is the correction due to the Stark effect. The parameter *a*_8_ > 0 determines the static susceptibility of a neutral acceptor and is known only for shallow acceptors in Si^22^: *a*_8_ ~ 10^−10^ eV cm^2^ V^−2^. Then an electric field strength as low as 10^3^ V cm^−1^ induces an energy shift of ~0.1 meV, which is comparable to the initial detuning Δ_lh_(0) − *E*_mp_. Hence, a relatively small electric field can control the exchange coupling constant. Substituting Eq. (6) into Eq. (5), we obtain

7 $${\mathrm{\Delta }}_{{\mathrm{pd}}}\left( E \right) = \frac{{\left| {H_{{\mathrm{ph}} - {\mathrm{h}}}} \right|^2}}{{{\mathrm{\Delta }}_{{\mathrm{lh}}}\left( 0 \right) - E_{{\mathrm{mp}}} + a_8E^2}}P_{{\mathrm{phon}}}^{\mathrm{c}}.$$

Since the static susceptibility *a*_8_ > 0^22^, our results demonstrate that the Δ_pd_ value is maximum for the case of flat bands (*E* = 0), where we have Δ_lh_(0) − *E*_mp_ > 0. The experiment^14^ shows that Δ_pd_ > 0 for *B*_F_ > 0, and therefore, as mentioned before, $P_{{\mathrm{phon}}}^{\mathrm{c}} > \,0$, i.e. the phonons are mainly σ^+^ polarized in agreement with Fig. 5.

We fit the data assuming that in a small range of reverse bias the electric field is proportional to the applied voltage $E \propto \left( {U - U_0} \right)$, where *U* = *U*_0_ corresponds to the flat band conditions, and drops entirely within the undoped region of ≤ 1 μm thickness (Fig. 1a). Thus from Eq. (7) we get a Lorentz curve

8 $$f\left( U \right) = \frac{A}{{1 + \left( {U - U_0} \right)^2/U_1^2}}$$

with halfwidth *U*_1_. The amplitude *A* gives the magnitude of the effect under flat bands conditions, and has different dimensions for the different experimental techniques. For SFRS the amplitude *A* in Eq. (8) gives the value of Δ_pd_(*U*) in μeV. The solid curve in Fig. 4c fits the results of the SFRS measurements well with *A* = 175 µeV, *U*_0_ = 0.7 V, and *U*_1_ = 1.8 V. For the polarization measurements the amplitude *A* in Eq. (8) is dimensionless. The solid line in Fig. 2d fits the polarization amplitude data in cw mode for *A* = 2.2%, *U*_0_ = 0.8 V, and *U*_1_ = 1.8 V. The time-resolved PL data of the polarization kinetics (also dimensionless) in Fig. 3b are described by *A* = 17%, *U*_0_ = 1.0 V, and *U*_1_ = 2.0 V. These fit parameters demonstrate good agreement. Therefore, the results of all three experimental techniques are explained within the model of the phononic ac Stark effect. Our results demonstrate that *U*_1_ ≈ 1.5 V is enough to switch the p–d interaction off, i.e. we obtain indeed a low-voltage control of magnetism.

## Discussion

At first glance the low-voltage control of the long-range exchange coupling looks surprising. Indeed, common sense suggests that application of an electric field in a direction that attracts holes inside the QW toward the FM should enhance the p–d exchange interaction. In contrast to that, the p–d exchange coupling decreases when reverse bias voltage (negative potential at the top electrode) is applied. This finding supports the earlier conclusions on the origin of the long-range p–d exchange interaction which is not related to the penetration of the electronic wavefunctions into the FM layer^14^. Our data rather demonstrate and confirm the suggested mechanism of the phononic ac Stark effect for electric field control of the exchange interaction, which is essentially different from traditional concepts. The coupling strength correlates with the band bending in the quantum well region and can be explained in the frame of the exchange coupling mediated by elliptically polarized phonons—the phononic ac Stark effect. The electric field changes the detuning of the heavy–light-hole energy splitting of the QW acceptor with respect to the magnon–phonon resonance energy in the FM.

While the present studies were performed at cryogenic temperatures, this condition likely does not represent a restriction for the coupling mechanism itself, but is merely caused by the detection through the QW optical properties (reduced values of spin-flip Raman signal and circular polarization degree of PL). Coherent propagation of acoustic phonons over micrometer distances at room temperature has been demonstrated^24^. Therefore, our results corroborate the feasibility of electrical control of the exchange interaction in hybrid ferromagnet-semiconductor nanostructures and can be potentially used for applications such as electric field effect magnetic memories. From a fundamental point of view, our achievement opens a principally different way for the control of magnetic interactions via the gate tunable phononic ac Stark effect that can be extended to various magnetic systems.

## Methods

### Sample fabrication

The sample of Co/(Cd,Mg)Te/CdTe/(Cd,Mg)Te/CdTe:I/GaAs (Fig. 1a) was grown on a (100)-oriented GaAs substrate by molecular-beam epitaxy. The buffer between the substrate and the quantum well is a 10 μm layer of conductive CdTe doped with iodine (donor concentration of the order of 10^18^ cm^−3^). The quantum well consists of a 0.5 μm wide (Cd,Mg)Te barrier layer, a 10 nm CdTe layer and an 8 nm (Cd,Mg)Te spacer. On top a 4-nm-thick cobalt layer was deposited. In order to make electrical contact to the CdTe:I buffer layer, a 5 mm in diameter mesa was etched into the structure to a depth of more than 0.8 μm. One contact is wired to the CdTe:I buffer layer, and the second contact is located at the cobalt surface.

### Continuous wave PL

For continuous wave polarization-resolved PL spectroscopy the sample was excited by the linearly polarized (π) light of a titanium-sapphire laser. In order to avoid sample heating the laser power was kept below 4 mW cm^−2^. The degree of circular polarization $\rho _{\mathrm{c}}^{\mathrm{\pi }} = \left( {I_ + ^\pi - I_ - ^\pi } \right)/\left( {I_ + ^\pi + I_ - ^\pi } \right)$ of the PL from the QW was detected, where $I_ + ^\pi$ and $I_ - ^\pi$ are the intensities of the σ^+^ and σ^–^ components with right and left circular polarization, respectively. The polarization degree $\rho _{\mathrm{c}}^{\mathrm{\pi }}$ does not depend on the orientation of the linear laser polarization. To magnetize the interfacial ferromagnet, a small magnetic field *B*_F_ (of the order of 100 mT) was applied in the Faraday geometry normal to the structure plane using a resistive magnet. The measurements were carried out at a temperature of 2 K.

### Time-resolved PL

Time-resolved PL allows one to obtain information about the transient processes of decay of the photoexcited carriers and their spin relaxation. Here, the sample is excited by short optical pulses with a central photon energy of 1.69 eV using a self-mode-locked titanium-sapphire laser with a repetition frequency of 75.75 MHz. The pulse duration was 150 fs, the spectral width of the laser was 10 nm, and the average pump density was ~4 W cm^−2^. The PL was dispersed with a 0.5 m focal length single monochromator to which a streak camera was attached for detection. The overall time resolution was about 20 ps. The experiments were carried out at a temperature of 2 K.

### Time-resolved pump–probe Kerr rotation

The coherent spin dynamics was measured by conventional time-resolved pump–probe Kerr rotation using a titanium-sapphire laser generating 1.5 ps pulses at the repetition frequency of 75.6 MHz (repetition period *T*_R_ = 13.2 ns). Electron spin coherence was generated along the growth *z*-axis of the sample by circularly polarized pump pulses. The polarization of the beam was modulated between σ^+^ and σ^−^ by a photo-elastic modulator operated at a frequency of 50 kHz. In order to avoid electron heating and delocalization effects the average pump density was kept at low levels ≤ 5 W cm^−2^. The probe beam was linearly polarized. The angle of its polarization rotation (*θ*_K_) or the ellipticity after reflection of the beam from the sample was measured by a polarization sensitive beamsplitter in conjunction with a balanced photodetector. Pump and probe beams had the same photon energy and were tuned to the energy of the exciton resonance. The sample was placed in the temperature insert of a vector magnet cryostat containing three superconducting split coils oriented orthogonally to each other. This magnet allows one to ramp the magnetic field up to 3 T and to carry out measurements with different orientations of the magnetic field relative to the sample at temperatures from *T* = 1.7 K up to 300 K.

### Spin-flip Raman scattering (SFRS)

The experiments were performed using resonant excitation with a cw laser at photon energies corresponding to the PL band of excitons bound to neutral acceptors (A^0^X) which is located about 1 meV below the exciton optical transition (see Fig. 2a). We used an oblique backscattering Faraday geometry where the excitation/detection beams and the magnetic field were parallel to each other, while the sample growth *z-*axis was tilted by 20° with respect to the magnetic field direction. The Raman shift was measured at a temperature of 2 K in magnetic fields up to 10 T in crossed circular polarizations for excitation and detection. The SFRS spectra were dispersed by a Jobin-Yvon U-1000 monochromator equipped with a cooled GaAs photomultiplier.

## Supplementary information

Supplementary information

## Data Availability

The data that support the plots within this paper and other findings of this study are available from the corresponding authors upon reasonable request.
