# Supplementary material for: Low voltage control of exchange coupling in a ferromagnet-semiconductor quantum well hybrid structure
Source: Nat Commun. 2019 Jul 1;10:2899. doi: 10.1038/s41467-019-10774-0 (PMC6603040; doi:10.1038/s41467-019-10774-0)
Supplement: Supplementary file 1 — Supplementary information [file 41467_2019_10774_MOESM1_ESM.pdf]

Supplementary material

Low voltage control of exchange coupling in a ferromagnet-semiconductor quantum well hybrid structure

V.L. Korenev[1*], I.V. Kalitukha[1], I.A. Akimov[1,2,*], V.F. Sapega[1], E.A. Zhukov[1,2], E. Kirstein[2], O.S. Ken[1], D. Kudlacik[2], G. Karczewski[3], M. Wiater[4], T. Wojtowicz[4], N.D. Ilyinskaya[1], N.M. Lebedeva[1], T.A. Komissarova[1], Yu.G. Kusrayev[1], D.R. Yakovlev[1,2], and M. Bayer[1,2]

[1]Ioffe Institute, Russian Academy of Sciences, 194021 St. Petersburg, Russia

[2]Experimentelle Physik 2, Technische Universität Dortmund, D-44227 Dortmund, Germany

[3]Institute of Physics, Polish Academy of Sciences, PL-02668 Warsaw, Poland

[4]International Research Centre MagTop, Institute of Physics, Polish Academy of Sciences PL-02668 Warsaw, Poland

Supplementary note 1. Pump-probe measurements in pulsed mode

Near the QW exciton resonance of the studied hybrid structure, pump-probe experiments performed in magnetic field are used to measure the frequency of the Larmor precession of conduction band electrons $\omega_e(B)$ and valence band holes $\omega_h(B)$ [1]. Under excitation with σ^+ polarized pump pulses, electrons and holes spin-polarized along the growth axis of the structure appear. Their spins precess about a perpendicular magnetic field as manifested by an oscillatory signal proportional to the *z*-component of the spins. Electrons and valence band holes precess with different frequencies due to the strong difference between their Landé *g*-factors. The electron *g*-factor in the CdTe QW is practically isotropic, while that of the valence band hole is strongly anisotropic [2]. Here, the experiment was performed in a tilted magnetic field, so that the field component along the sample growth direction (*z*-axis) induces the magnetization of the interfacial FM, while its transverse component causes spin precession of the charge carriers in the QW. The details are described in Ref. [1]. We monitor the precession of the valence band hole spins induced by the magnetic field for $U = +1$ V. The measurements were carried out in magnetic fields of 0.5–3 T tilted by $\theta = 80°$ with respect to the *z*-axis, so that its *z*-component was in the range of 100–520 mT. This is sufficient to magnetize the interfacial FM along the easy *z*-axis and to cause the FM proximity effect [3]. An example of spin oscillations in a magnetic field of $B_V = 1$ T in the Voigt geometry is shown in Supplementary Figure 1a. A superposition of electron (fast) and hole (slow) oscillations is observed. Fitting with two exponentially damped harmonic functions allows one to determine the oscillation frequencies. The magnetic field dependences of the hole Zeeman splitting $\hbar\omega_h(B)$ for $\theta = 80°$ (red circles) and 90° (blue circles) are plotted in the Supplementary Figure 1b.

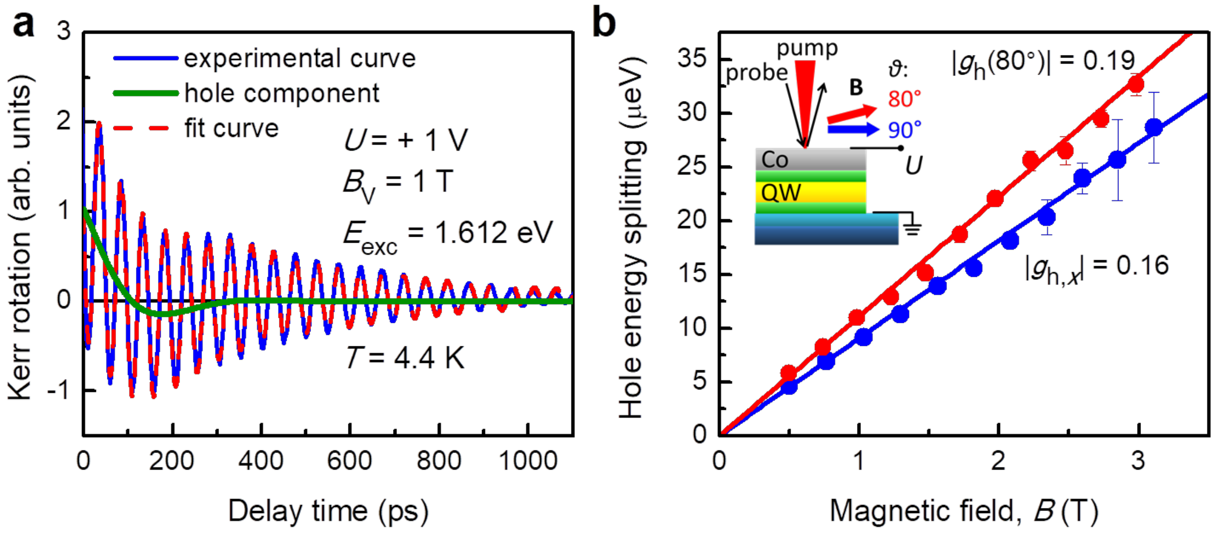

Supplementary Figure 1. (a) Oscillations of the Kerr-rotation angle in a magnetic field of $B_V = 1$ T in the Voigt geometry for a forward bias $U = +\ 1$ V. (b) Dependence of the Zeeman splitting of the hole on the magnetic field for the Voigt geometry ($\theta = 90°$, blue circles) and for a tilted field ($\theta = 80°$, red circles). The voltage $U = +\ 1$ V, the photon energy of the pump and the probe is 1.612 eV, the power density of both the pump and the probe is 5 W cm^{-2}. $T = 4.4$ K. Error bars represent standard deviations.

Supplementary Figure 1b shows that the g-factor of the hole is anisotropic: $|g_{h,x}| = 0.16$, $\left|g_\mathrm{h}(\theta = 80^\circ)\right| = \sqrt{g_{\mathrm{h},z}^2 \cos^2\theta + g_{\mathrm{h},x}^2 \sin^2\theta} = 0.19$. Hence we obtain $|g_{h,z}| = 0.66$. Extrapolation of the dependence $\hbar\omega_\mathrm{h}(B)$ to zero field does not show any offset for the valence band hole, just as it was the case in the analogous structure without contacts [1]. This indicates that the exchange interaction of the valence band holes with the ferromagnet is negligible, which is in agreement with the data in Fig. 2b (stars). Also in the magnetic field dependence of the electron Zeeman splitting $\hbar\omega_\mathrm{e}(B)$ no offset is observed (not shown). Thus, neither the valence band hole, nor the electron experience an exchange interaction with the ferromagnet.

Supplementary note 2. Ferromagnetic proximity effect at forward bias

Here we show that the decrease of the FM proximity effect for forward bias in the cw as well as time-resolved PL and the SRFS experiments is related with the appearance of valence band holes in the quantum well. The situation is illustrated in Supplementary Figure 2. The band structure diagram in equilibrium ($U = 0$) is shown in Supplementary Figure 2a. The holes fill the acceptor states, while holes in the valence band are absent. Application of a positive voltage $U > +0.5$ V flattens the bands, so that a fraction of holes moves from interface states to the valence band in the QW (Supplementary Figure 2b). A further increase of U does not lead to band bending because the voltage is distributed throughout the structure, and the electric field in the QW region is small in accordance with the PL spectra. This picture is confirmed by all our experiments, as we will now discuss. SFRS gives the clearest proof of the appearance of valence band holes in the QW for forward bias.

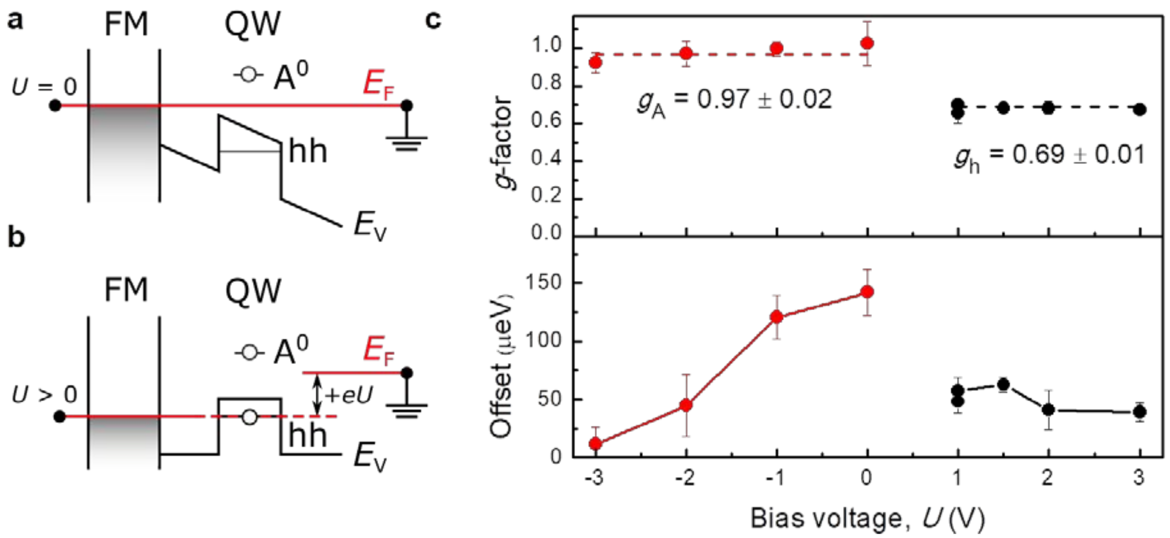

Supplementary Figure 2. Section of the hybrid structure band diagram in equilibrium (a) and for forward bias (flat band conditions) (b). The Fermi level E_F, the energy of the neutral acceptor A^0, and the lowest QW energy level of the heavy holes in the valence band (hh) are shown schematically. (c) Hole g-factor (top) and offset energy (bottom) dependences on the bias voltage, measured by SFRS. The magnetic field is tilted by 20° with respect to the sample growth z-axis, $T = 2$ K. The dashed lines are guidelines for the eye. Error bars represent standard deviations.

In addition to the offset, SFRS gives the hole *g*-factor (of the valence band hole or of the hole bound to an acceptor). When the A^0X transition is excited, a spin flip of the acceptor hole can occur. From the slope of the linear fit to the magnetic field dependence of the Raman shift for reverse biases (see Fig. 4c) one calculates the *g*-factor of the hole bound to an acceptor to be $|g_A| = 0.97 \pm 0.02$. However, for forward bias the hole *g*-factor changes to 0.69 (Supplementary Figure 2c, top), and the offset value decreases from 140 to 50 μeV (bottom). Interestingly, the *g*-factor of 0.69 ± 0.01 is close to the value of the longitudinal *g*-factor of the valence band hole $|g_{h,z}| = 0.66$, as determined from the pump-probe measurements (see Supplementary Figure 1a). These results indicate the contribution of valence band holes to SFRS for $U > +0.5$ V, in addition to holes bound to acceptors, in accordance with Supplementary Figure 2a. The presence of holes in the valence band enables one to excite positively charged excitons (X^+ trion), quasiparticles consisting of two band holes and an electron, in the SFRS experiment. The binding energies of the A^0X complex and the X^+ trion are close to each other. According to the Haynes' rule the A^0X binding energy is about 10% of the acceptor hole binding energy [4], i.e. about 4 meV. In turn, the X^+ trion binding energy in the CdTe QW is 3–4 meV [5]. Therefore, PL excitation spectra of the A^0X and X^+ optical transitions overlap and so do the spin flips of A^0 and free holes. However, we are able to cancel out the X^+ contribution because the gate voltage controls their concentration ratio. The probabilities of the A^0X and X^+ optical transitions are proportional to the concentrations of A^0 acceptors and valence band holes in the QW, respectively. For $U < +0.5$ V the valence band is empty of holes, so that only the A^0X transition is excited. In this case we observe unambiguously exchange coupling of the FM with the A^0 holes and detect the g-factor of A^0 and a large offset. However, for $U > +0.5$ V holes appear in the QW valence band and X^+ transitions contribute to spin-flip Raman process (according to the same mechanisms as in the acceptor hole spin flip in Supplementary Figure 3), thus changing the slope of the magnetic field

dependence of the Raman shift and decreasing the offset value at zero magnetic field. Since the p-d exchange with the band hole is much smaller than that with the hole bound to an acceptor, the measured Δ_{pd} value decreases.

The presence of valence band holes also explains the sharp decrease of the FM proximity effect with increasing voltage, followed by saturation for $U > +0.5$ V, as observed in the polarization-resolved cw (Fig. 2d) and time-resolved PL (Fig. 3b) recorded on the e-A^0 line. The corresponding Equations (1) and (2) for the polarization of the holes bound to acceptors (the e-A^0 line) are valid only in the absence of valence band holes. Scattering with a mutual spin-flip (flip-flop transition) of the holes on neutral acceptors will lead to an averaging of the hole polarization in different orbital states. This mechanism of averaging is well known for conduction band electrons that scatter on electrons bound to neutral donors [6]. The valence band holes interact weakly with the FM, and therefore the average polarization of the hole spin system will be reduced due to the spin exchange.

Thus, the results of all four experiments (cw and time-resolved PL, pump-probe Kerr rotation, SFRS) are consistent with each other, if we take into account the appearance of valence band holes in the QW (in addition to A^0 holes) for forward bias. The valence band holes do not appear due to electrical injection, because the effects saturate and do not scale exponentially with voltage like the correspondingly increasing current. Photo-injection of the holes can be excluded as well, since the SFRS measurements were carried out under resonant excitation below the interband and exciton absorption, and the behavior of the polarization dependences (see Figs. 2d and 3b) does not change with light intensity (at excitation power levels ≤ 5 W cm^{-2}). The most probable reason for the appearance of valence band holes is charging of the quantum well in darkness, as illustrated in Supplementary Figure 2a, b.

Supplementary note 3. Spin-flip Raman scattering on neutral acceptor

Here, we consider the main processes of the spin flip of a hole bound to an acceptor. We use Faraday geometry (direction of excitation and scattered light are parallel to the magnetic field) while the sample is tilted by a small angle $\theta << 1$ ($\cos\theta \approx 1$) between the magnetic field direction and the *z*-axis. This relaxes the selection rules which are dictated by angular momentum conservation due to mixing of the electron states with spin projections of +1/2 and -1/2 onto the *z*-axis (↑ and ↓, respectively, as indicated in Supplementary Figure 3). The mixing parameter is given by $\beta \approx \theta/2$. In all cases the resonant intermediate (virtual) state is given by the exciton complex bound to a neutral acceptor (A^0X).

Supplementary Figure 3a shows the double spin flip (DSF) scattering process [7]. This scattering involves a spin flip of the electron in the photoexcited exciton which is accompanied by emission of an acoustic phonon whose energy $\hbar\omega_q$ is equal to the Zeeman splitting $\mu_B|g_e|B$ of the conduction band electron in the A^0X complex. In the initial state, a σ^- photon comes in with energy $\hbar\omega_1$ (tuned to the A^0X optical transition) and the acceptor has an angular momentum projection +3/2 as indicated by ⇑ in Supplementary Figure 3. In the final state, there are a σ^+ photon with energy $\hbar\omega_2$, the phonon with energy $\hbar\omega_q$, and an acceptor with momentum projection -3/2. It follows from energy conservation that the Stokes shift for the double spin flip $\Delta_S^{DSF} = \hbar\omega_1 - \hbar\omega_2 = \mu_B(|g_e| + |g_A|)B$ is determined both by the *g*-factor of the electron (g_e) and of the acceptor-bound hole (g_A). In the presence of p-d exchange interaction, there is an additional contribution $-\Delta_{pd}$ to the Stokes shift, so that we arrive at Equation (3).

The single spin flip (SSF) scattering process is shown in Supplementary Figure 3b. Here, a phonon assisted spin flip of the conduction band electron in the excited state is not required. In presence of the p-d exchange interaction, the Stokes shift for the SSF is given by Equation (4),

which is determined only by the g-factor of the hole on the acceptor and by the exchange splitting. This process was observed in Ref. [1], which allowed us to directly measure Δ_{pd}. Both the DSF and SSF mechanisms occur in the structure studied here (see Fig. 4a, lines ′e+h′ and ′h′, respectively). The change in the angular momentum of the photons by 2 quanta is analyzed using crossed circular polarizer and analyzer for both mechanisms.

Finally, there is a third spin-flip process, which corresponds to the single electron spin flip accompanied by emission of an acoustic phonon with energy $\hbar\omega_q = \mu_B |g_e| B$ in the A^0X complex. It becomes allowed due to the B-field induced mixing of the states +1/2 and -1/2. This mechanism corresponds to the ′e′ line in the SFRS spectrum in Fig. 4a.

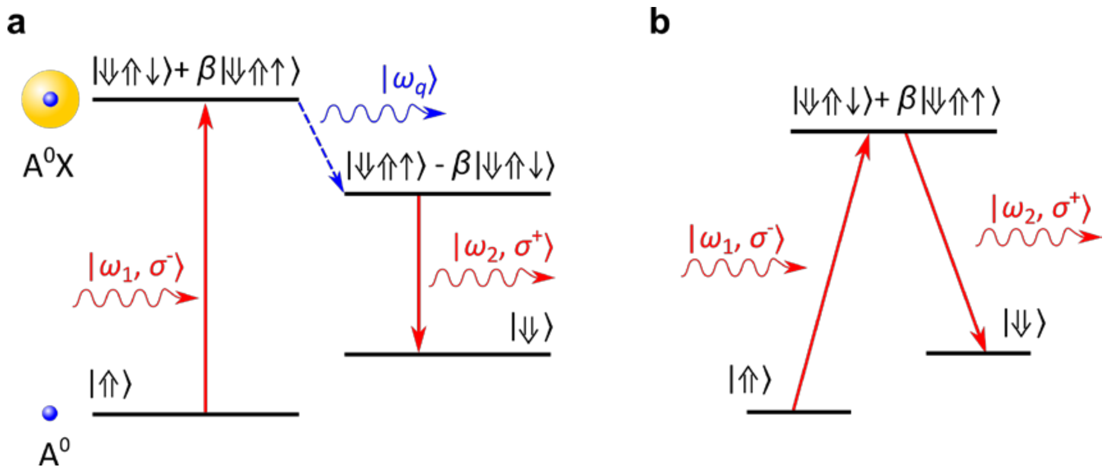

Supplementary Figure 3. Energy diagram of SFRS in a magnetic field $B > \Delta_{pd} / \mu_B |g_A|$ (a) Scheme of the double spin flip (DSF). Here, ⇑, ⇓ indicate the angular momentum projections of the acceptor J_z = +3/2, -3/2 and ↑, ↓ the electron spin projections s_z = +1/2, -1/2 onto the z-axis. (b) Scheme of the single spin flip (SSF).

Supplementary references:

[1] Akimov, I. A., Salewski, M., Kalitukha, I. V., Poltavtsev, S. V., Debus, J., Kudlacik, D., Sapega, V. F., Kopteva, N. E., Kirstein, E., Zhukov, E. A., Yakovlev, D. R., Karczewski, G.,

Wiater, M., Wojtowicz, T., Korenev, V. L., Kusrayev, Yu. G., and Bayer, M. Direct measurement of the long-range p-d exchange coupling in a ferromagnet-semiconductor Co/CdMgTe/CdTe quantum well hybrid structure. *Phys. Rev. B* **96**, 184412 (2017).

[2] Sirenko, A. A., Ruf, T., Cardona, M., Yakovlev, D. R., Ossau, W., Waag, A., and Landwehr, G. Electron and hole g factors measured by spin-flip Raman scattering in CdTe/CdMgTe single quantum wells. *Phys. Rev. B* **56**, 2114 (1997).

[3] Korenev, V.L., Salewski, M., Akimov, I.A., Sapega, V.F., Langer, L., Kalitukha, I.V., Debus, J., Dzhioev, R.I., Yakovlev, D.R., Mueller, D., Schroeder, C., Hoevel, H., Karczewski, G., Wiater, M., Wojtowicz, T., Kusrayev, Yu.G., and Bayer, M. Long-range p-d exchange interaction in a ferromagnet-semiconductor hybrid structure. *Nature Physics* **12**, 85 (2016).

[4] Haynes, J.R. Experimental Proof of the Existence of a New Electronic Complex in Silicon *Phys. Rev. Lett.* **4**, 361 (1960).

[5] Debus, J., Dunker, D., Sapega, V. F., Yakovlev, D. R., Karczewski, G., Wojtowicz, T., Kossut, J., and Bayer, M. Spin-flip Raman scattering of the neutral and charged excitons confined in a CdTe/(Cd,Mg)Te quantum well. *Phys. Rev. B* **87**, 205316 (2013).

[6] Paget, D. Optical detection of NMR in high-purity GaAs under optical pumping: Efficient spin-exchange averaging between electronic states. *Phys. Rev. B* **24**, 3776 (1981).

[7] Sapega, V. F., Ruf, T., Cardona, M., Ploog, K., Ivchenko, E. L., and Mirlin, D. N. Resonant Raman scattering due to bound-carrier spin flip in GaAs/AlGaAs quantum wells. *Phys. Rev. B* **50**, 2510 (1994).
